# Prolactin Levels Correlate with Abnormal B Cell Maturation in MRL and MRL/lpr Mouse Models of Systemic Lupus Erythematosus-Like Disease

**DOI:** 10.1155/2013/287469

**Published:** 2013-12-10

**Authors:** Maria Victoria Legorreta-Haquet, Rocio Flores-Fernández, Francisco Blanco-Favela, Ezequiel M Fuentes-Pananá, Luis Chávez-Sánchez, Rafael Hernández-González, Emiliano Tesoro-Cruz, Lourdes Arriaga-Pizano, Adriana Karina Chávez-Rueda

**Affiliations:** ^1^UIM en Inmunología, Hospital de Pediatría, CMN Siglo XXI, IMSS, 06720 Mexico City, DF, Mexico; ^2^Departamento de Inmunología, ENCB, IPN, 11340 Mexico City, DF, Mexico; ^3^Unidad de Investigación en Virología y Cáncer Hospital Infantil de México Federico Gómez, 06720 Mexico City, DF, Mexico; ^4^Departamento de Investigación Experimental y Bioterio del Instituto Nacional de Ciencias Médicas y Nutrición “Salvador Zubirán”, 14000 Mexico City, DF, Mexico; ^5^UIM en Inmunoquímica, Hospital de Especialidades, CMN Siglo XXI, IMSS, 06720 Mexico City, DF, Mexico

## Abstract

Prolactin (PRL) plays an important role in modulating the immune response. In B cells, PRL enhances antibody production, including antibodies with self-specificity. In this study, our aims were to determine the level of PRL receptor expression during bone-marrow B-cell development and to assess whether the presence of high PRL serum concentrations influences absolute numbers of developing populations and disease outcome in lupus-prone murine models. We observed that the PRL-receptor is expressed in early bone-marrow B-cell; the expression in lupus-prone mice, which had the highest level of expression in pro-B cells and immature cells, differed from that in wild-type mice. These expression levels did not significantly change in response to hyperprolactinemia; however, populations of pro-B and immature cells from lupus-prone strains showed a decrease in the absolute numbers of cells with high PRL-receptor expression in response to PRL. Because immature self-reactive B cells are constantly being eliminated, we assessed the expression of survival factor BIRC5, which is more highly expressed in both pro-B and immature B-cells in response to PRL and correlates with the onset of disease. These results identify an important role of PRL in the early stages of the B-cell maturation process: PRL may promote the survival of self-reactive clones.

## 1. Introduction

Prolactin (PRL) is predominantly produced by the lactotropic cells of the anterior pituitary gland. However, it is also generated in extrapituitary sites, such as immune, decidual, mammary, epithelial, and fat cells [1–3]. PRL has multiple regulatory roles in reproduction, development, growth, osmosis, metabolism of carbohydrates and lipids, and the immune response. The PRL receptor is a member of the cytokine receptor superfamily [3–5]. Different isoforms of the PRL receptor have been found to be generated by alternative splicing at the 3′ end and variation in the intracellular domain length [3, 5, 6]. The PRL receptor is expressed in many immune cell types, mainly B cells, and also T cells, monocytes, macrophages, natural killer (NK) cells, and thymic epithelial cells [7, 8], and its activation induces transcriptional programs involved in various cellular functions such as proliferation, differentiation, and cytokine production. Hence, PRL has been implicated as a modulator of both cellular and humoural immunity [8–11]. 

Elevated serum PRL levels have been reported in several autoimmune diseases, including systemic lupus erythematosus (SLE) [12–14]. SLE is an autoimmune rheumatic disease. Serum samples from SLE patients characteristically have very strong reactivity to a variety of nuclear components, including DNA, RNA, histones, RNP, Ro and La. These antibodies form immune complexes that are deposited in the kidneys and may result in proteinuria and kidney failure. The presence of these autoantibodies indicates abnormalities in the activation and development of B cells [15, 16] and both B and T cells express the PRL receptor and secrete PRL [4, 17, 18]. SLE affects women of reproductive age at a 9 : 1 ratio compared to men and this gender bias has been attributed to the immunostimulatory properties of hormones. SLE symptoms typically begin or become exacerbated during pregnancy, when PRL serum levels are high. Nonphysiologically high serum concentrations of PRL also correlate with SLE symptoms [12, 14]. These findings have been reproduced in murine models of lupus (e.g., (NZB × NZW)F1 and MRL/lpr), in which the induction of hyperprolactinemia correlated with exacerbated disease symptoms, such as the early detection of autoantibodies, proteinuria and accelerated death [19, 20]. MRL-MpJFas^lpr^ (MRL/lpr) mice have a mutation in the Fas gene and develop a disease similar to SLE that is characterised by glomerulonephritis, vasculitis, splenomegaly, hypergammaglobulinemia, and the production of anti-dsDNA antibodies [21]. In this mouse strain, B cell elimination using an anti-CD79 antibody decreased the manifestation of SLE-like symptoms, demonstrating the importance of B cells in SLE physiopathology [22, 23].

B cells develop from hematopoietic stem cells in the bone marrow through a series of differentiation stages. The most immature cell committed to the B cell lineage is the B cell progenitor, also called the pro-B cell, which undergoes immunoglobulin heavy chain gene rearrangement and differentiates into a pre-B cell. Pre-B cells undergo immunoglobulin light chain gene rearrangement and develop into immature B cells. This latter population is tested for self-specificity first in bone marrow then in circulation and the spleen, where it is identified as transitional type I (T-1) B cells. These cells further develop into transitional type II (T-2) and type III (T-3) B cells to finally become mature B cells (follicular, and marginal zone cells) [24–27]. B cell antigen receptor assembly and testing for autoreactivity are the primary objectives of B cell development; therefore, the alteration of this maturation pathway results in a generation of B cell clones with the potential to cause autoimmune diseases.

Our group previously demonstrated that T-1 B cells express the highest level of PRL receptor of any other splenic B cell population. We also observed a significant increase in the absolute number of this B cell subset in mice that developed lupus during hyperprolactinemia [20]. Because T-1s represent the first subset of splenic B cells produced by bone marrow cells, it is possible that PRL targets earlier bone marrow developmental stages. Therefore, the aim of this study was to determine whether developing bone marrow B cells express the PRL receptor and whether development is altered in response to PRL sera levels that correlate with the onset of lupus in a murine model of this disease. Our results showed that all early bone marrow B cell populations express the PRL receptor. However, the expression was higher in pro-B and immature cells in lupus-prone mice, a pattern that differs from that of wild type mice. Increased levels of PRL hastened disease manifestations, which correlated with a reduction in the absolute number of maturing B cells. These results support an important role of PRL in the early stages of the B cell maturation process, thus helping to clarify its relevance to the development of SLE. 

## 2. Materials and Methods

### 2.1. Mice

All studies were approved by the Animal Care Committee of Instituto Nacional de Ciencias Médicas y Nutrición “Salvador Zubiran” and Hospital de Pediatría, Centro Médico Nacional Siglo XXI, IMSS (R-2011-785-015), and all of the mice experiments were performed in accordance with approved guidelines established by Mexico (Norma Oficial Mexicana NOM-062-ZOO-1999). The C57BL/6 mice were purchased from Harlan (Indianapolis, USA); the MRL/MpJ (MRL) and MRL/MpJFas^lpr^ (MRL/lpr) mice were purchased from The Jackson Laboratory (Maine, USA). Mice were housed in a pathogen-free barrier facility and were provided with sterile food and water *ad libitum*. 

### 2.2. Antibodies

The following antibodies were used: APC-conjugated rat anti-mouse CD21 (7G6) from BD Biosciences (Mountain View CA, USA); FITC-conjugated rat anti-CD43 (eBioR2160), PE-conjugated rat anti-B220 (RA3-6B2), APC-conjugated rat anti-IgM (11/41), PE/Cy7-conjugated rat anti-CD23 (B3B4), PE-conjugated rat anti-CD93 (AA4.1), and FITC-conjugated rat anti-CD19 (eBioD3) from eBioscience (San Diego, CA, USA); goat anti-mouse PRL-R (E20) from Santa Cruz Biotechnology (Santa Cruz, CA, USA), and biotinylated swine anti-goat from Invitrogen (Carlsbad CA, USA). The biotinylated secondary antibody was detected using phycoerythrin-Cy5.5 conjugated streptavidin from BD Biosciences (Mountain View, CA, USA). AffiniPure F(ab)_2_ fragment goat anti-mouse IgM was from Jackson ImmunoResearch (Baltimore, USA).

### 2.3. Purification of B Cells

Bone marrow (BM) cells were collected by flushing femoral shafts with cold RPMI (HyClone, Logan, Utah, USA) supplemented with 2% bovine serum albumin (BSA, US Biological, Swampscott, Ma, USA) and EDTA 2 mM (IBI Scientific, USA). After red blood cell depletion using lysis buffer (Sigma Aldrich, St. Louis, Missouri, USA), the cells were incubated with anti-B220 microbeads (Miltenyi Biotec, Bergisch Gladbach, Germany), and B cells were isolated by positive selection using a magnetic activated cell-sorting (MACS) system (Miltenyi Biotec, Bergisch Gladbach, Germany). After purification, >98% of the remaining cells were CD19^+^ by flow cytometry.

### 2.4. Cell Sorting

B cells suspensions from BM were incubated with fluorescently labelled antibodies specific for CD43, B220, IgM, and CD23 in staining buffer (PBS with 0.5% BSA) for 20 minutes at 4°C. The cells were washed, and the B cell (B220^+^) subsets were separated according to the expression of the following surface markers: pro-B (CD43^+^, CD23^−^, and IgM^−^), pre-B (CD43^−^, CD23^−^, and IgM^−^), and immature cells (CD43^−^, CD23^−^, IgM^+^). Cell sorting was performed using a FACSAria sorter with FACSDiva software (BD Bioscience, Mountain View, CA, USA). The purity of the sorted cells ranged from 95% to 98%.

### 2.5. Real Time RT-PCR

Total RNA was extracted from B cells using TRIzol reagent (Invitrogen, Carlsbad, CA, USA) according to the manufacturer's protocol and the RNA concentration was determined using UV spectrophotometry. SuperScript II Reverse Transcriptase (Invitrogen, Carlsbad, CA, USA) was used to generate cDNA from 1 *μ*g of total RNA according to the manufacturer's specifications. Genes of interest were amplified and quantified by real time RT-PCR using the LightCyclerTaqMan Master kit (Roche Diagnostic, Mannheim, Germany) according to the manufacturer's specifications. Hydrolysis probes and primers were designed by Roche Diagnostic. The following primers were used: PRL receptor 5′-CAGTAAATGCCACGAACGAA-3′ (left), PRL receptor 5′-GAGGAGGCTCTGGTTCAACA-3′ (right), *β*-actin 5′-AAGGCCAACCGTGAAAAGAT-3′ (left), *β*-actin 5′-GTGGTACGACCAGAGGCATAC-3′ (right), BIRC5 (survivin) 5′-CCCGATGACAACCCGATA-3′ (left) and BIRC5 5′-CATCTGCTTCTTGACAGTGAGG-3′ (right). The final reaction volume was 10 *μ*L. A LightCycler Instrument (Roche Diagnostic, Mannheim, Germany) was used to perform the RT-PCR reaction. The following RT-PCR conditions were used: 10 minutes at 95°C, followed by 40 cycles of 10 seconds at 95°C, 30 seconds at 60°C and 1 second at 72°C and 1 cycle of 30 seconds at 40°C. The *β*-actin gene was used as a normalisation control across samples. The relative expression of the PRL receptor and BIRC5 were calculated using the 2-ΔCT formula.

### 2.6. Induction of Hyperprolactinemia

Nine-week-old C57BL/6, MRL, and MRL/lpr mice (8 females per group) were subcutaneously injected with 200 *μ*g of metoclopramide (Sigma Aldrich, St. Louis, MO, USA) in 100 *μ*L of PBS for six weeks. A matched control group (C57BL/6, MRL and MRL/lpr) received PBS only (100 *μ*L) over the same period. Urinary protein levels were assessed semiquantitatively using reagent strips for urinalysis (UriCheck-10, Axilab, Monterrey, NL, Mex). Serum samples obtained at the beginning and at the end of the experiments were kept at −35°C until they were assayed for PRL and anti-dsDNA antibodies.

### 2.7. Prolactin Assessment

Serum levels of PRL were detected by ELISA by coating 96-well MaxiSorp plates (Nunc, Rochester, NY, USA) overnight with 100 *μ*L of 2 *μ*g/mL anti-mouse PRL monoclonal antibody (clone 207518, R&D Systems, Minneapolis, MN, USA) in PBS at 4°C, block with 2% BSA, and incubat with the serum sample (1 : 10) overnight at 4°C. Recombinant mouse PRL (National Hormone and Peptide Program, NIH, donated by AF Parlow) was used as a standard. The plates were then incubated with 0.2 *μ*g/mL biotinylated anti-PRL antibody (R&D Systems, Minneapolis MN, USA), avidin-conjugated alkaline phosphatase (Invitrogen, Carlsbad, CA, USA) and the enzyme substrate 5-bromo-4-chloro-3-indolyl phosphate (Sigma-Aldrich, St. Louis MO, USA) according to the manufacturer's instructions. The OD was measured at 405 nm using a Dynatech MR5000 ELISA reader.

### 2.8. Measurement of Anti-dsDNA Antibodies

Anti-dsDNA antibody serum concentrations were detected using ELISA. A 96-well MaxiSorp plate was coated with 100 *μ*L of 5 *μ*g/mL calf thymus dsDNA (Sigma Aldrich, St. Louis MO, USA) in bicarbonate buffer overnight at 4°C and was blocked with 2% BSA. The plates were then incubated for 1 h at 37°C with serum (1 : 50) or the anti-dsDNA antibody standard (clone 16-13, Chemicon International, Billerica MA, USA), followed by alkaline phosphatase-conjugated rabbit anti-mouse IgG (Invitrogen, Carlsbad, CA, USA) and substrate (5-bromo-4-chloro-3-indolyl phosphate). The OD was monitored at 405 nm using a Dynatech MR5000 ELISA reader. 

### 2.9. Cell Surface Staining and Flow Cytometry

BM cells were incubated with fluorescently labelled antibodies for 20 minutes at 4°C in staining buffer (PBS with 0.5% BSA and 0.01% sodium azide). The cells were then washed and fixed in 2% paraformaldehyde (Sigma Aldrich, St Louis MO, USA). The data were acquired using a FACSAria flow cytometer and analysed with FlowJo software (Tree Star, Ashland, OR, USA).

### 2.10. Statistical Analysis

The data were analysed using standard statistical tests (mean value, SD, Student's *t*-test, and ANOVA) and the results are expressed as the mean ± SD. The level of significance was set at *P* ≤ 0.05. All calculations were performed using SPSS 19 software.

## 3. Results

### 3.1. Expression of the PRL Receptor in B Cells

**Figure 1:**
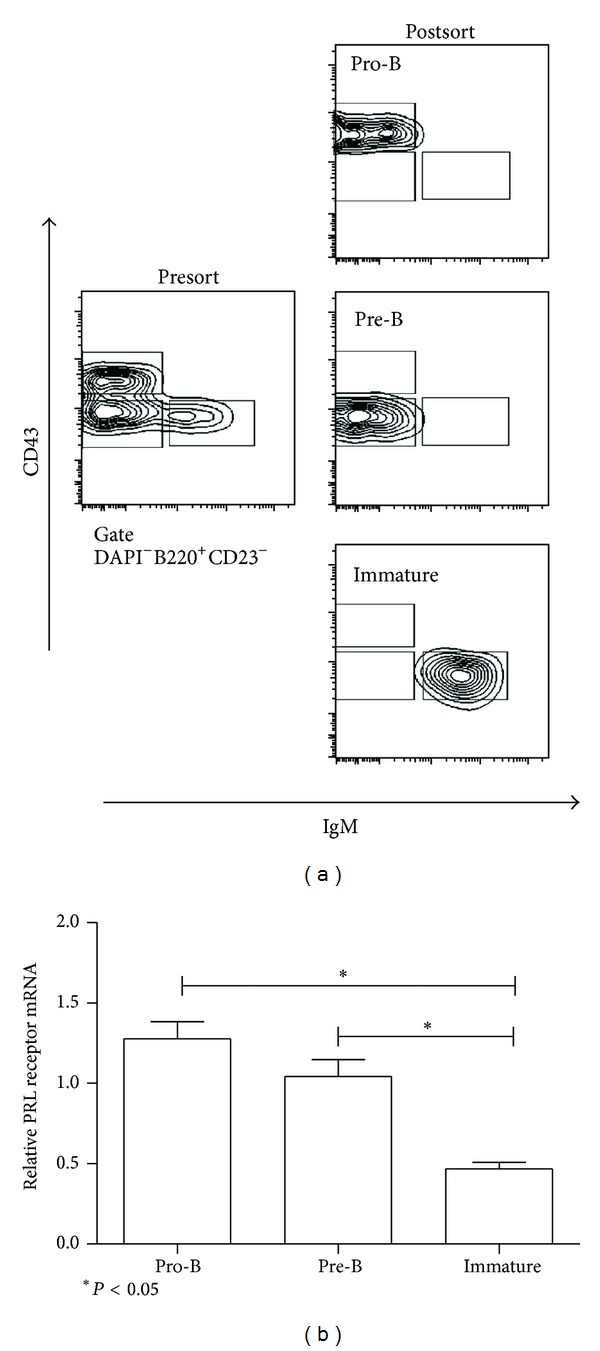
Purification of B cell by flow cytometry. B cells were purified from the BM of 9-week-old mice. (a) The cells were incubated with antibodies specific for B cell subsets, and the subsets were purified using flow cytometry, as detailed in Methods. The purity of the collected populations varied between 95% and 98%. A representative example of the purified B cells from wild-type C57BL/6 mice is shown. (b) Using RT-PCR, the PRL receptor mRNA expression was measured in the different subsets of bone marrow B cells (pro-B, pre-B and immature). The asterisks denote statistical significance with the *P* value shown.

**Figure 2:**
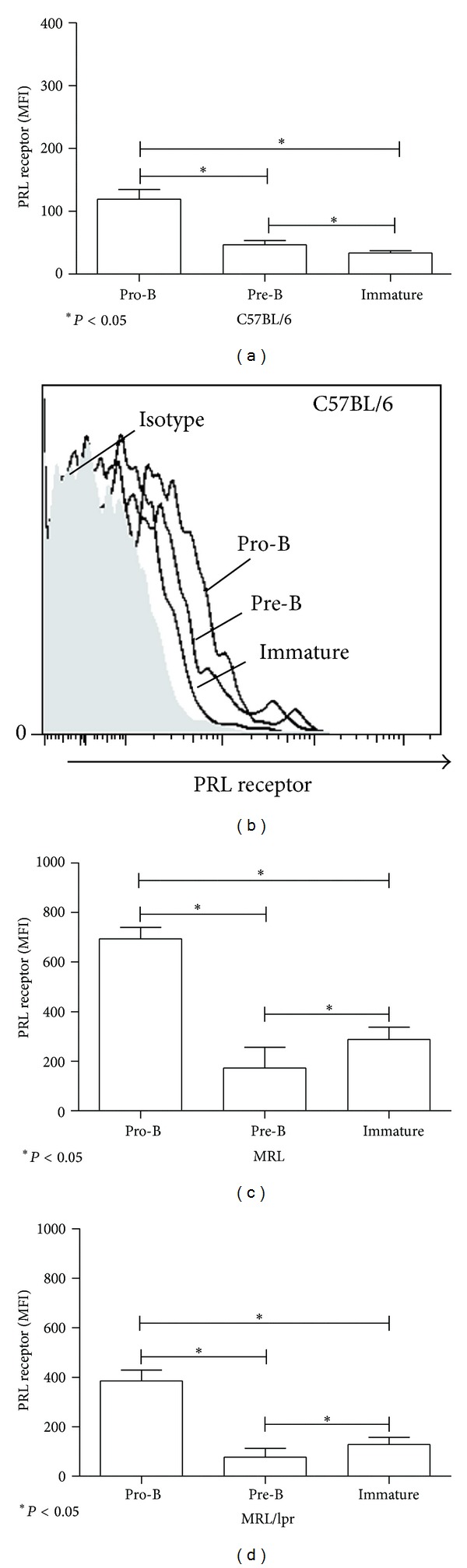
Expression of prolactin receptor in B cells. PRL receptor expression (MFI) was measured using flow cytometry from eight mice per strain. BM cells were labelled with anti-B220, anti-CD43, anti-CD23, anti-IgM, and goat anti-PRL receptor antibodies; the isotype control was labelled with anti-B220, anti-CD43, anti-CD23, anti-IgM, and goat unrelated antibodies. (a) Pro-B, pre-B and immature from C57BL/6 mice; (b) histograms of PRL receptor expression in B cells from BM; (c) pro-B, pre-B, and immature from MRL mice; (d) pro-B, pre-B, and immature from MRL/lpr mice. The asterisks denote statistical significance with the *P* value shown. The MFI values expressed in the graphs correspond to the MFI values minus the isotype control.

The pro-B, pre-B, and immature B cells from the bone marrow of C57BL6 wild-type mice were purified by flow cytometry to >95% purity (Figure 1(a)) and were assayed for the expression of PRL receptor mRNA and protein. Our results showed that all B cell developmental stages in the bone marrow express the PRL receptor. Immature B cells had the lowest relative mRNA expression (0.47 ± 0.04), which was significantly different (*P* < 0.05) compared to pre-B cells (1.04 ± 0.18) and pro-B cells (1.28 ± 0.10); there was no significant difference between pro-B and pre-B cells (Figure 1(b)). A similar expression pattern was observed at the protein level; immature B cells had the lowest PRL receptor expression (35.77 ± 9.41 MFI, mean fluorescence intensity), followed by pre-B cells (46.67 ± 6.05 MFI) and pro-B cells (119.30 ± 42.51 MFI) (Figure 2(a)). Thus, PRL receptor expression of pro-B cells is 2.6 times higher than that of pre-B cells and 3.3 times higher than that of immature cells.  Figure 2(b) shows the flow cytometry histograms. 

### 3.2. Receptor Expression in Lupus-Prone Mice

Analysis of BM B cells from both lupus-prone strains (MRL and MRL/lpr) at 9 weeks of age (without disease manifestations) revealed a different PRL receptor expression pattern; pro-B cells had the highest level of PRL receptor expression (4.5- to 5.5-fold more than pre-Bs and 2.4- to 3-fold more than immature B cells). Thus, in lupus-prone mice, pro-B cells were followed by immature and pre-B cells as shown in Figure 2(c) (MRL: pro-B = 693.60 ± 46.56 MFI, pre-B = 153.40 ± 37.67 MFI, and immature = 288.00 ± 58.85 MFI) and Figure 2(d) (MRL/lpr: pro-B = 385.33 ± 43.70 MFI, pre-B = 77.66 ± 35.74 MFI, and immature = 128.50 ± 28.73 MFI); the differences between all populations were statistically significant. In this analysis, MRL mice showed the highest receptor expression, followed by MRL/lpr and C57BL/6 in all BM B cell populations. 

### 3.3. Exacerbation of SLE by Hyperprolactinemia

Nine-week-old MRL/lpr, MRL, and C57BL/6 mice were treated with metoclopramide for six weeks to induce high levels of PRL and accelerate SLE symptoms. The serum concentrations of PRL for pretreatment (9 weeks), PBS-treated, and metoclopramide-treated mice were 4.2 ± 1.38, 3.80 ± 1.18, and 10.70 ± 1.23 ng/mL, respectively, for the C57/BL6 strain, 12.58 ± 1.99, 11.20 ± 1.81, and 26.27 ± 2.69 ng/mL, respectively, for the MRL strain, and 12.73 ± 2.25, 20.07 ± 2.75 and 34.51 ± 4.34 ng/mL, respectively, for the MRL/lpr strain. All strains had significantly increased PRL levels in sera in response to metoclopramide (hyperprolactinemia), while only MRL/lpr mice showed a significant increase after PBS treatment, which was likely age-related (15 weeks at the end of treatment); however, the PRL increase in the PBS group was lower than in the group treated with metoclopramide (Table 1). 

Proteinuria and serum anti-dsDNA antibodies, two disease manifestations that mirror lupus symptoms, were measured and the concentrations were compared between MRL and MRL/lpr mice before and after treatment with metoclopramide or PBS. All mice had a significant increase in proteinuria in response to metoclopramide, while only the PBS-treated group of MRL/lpr mice also showed a significant increase correlating with the observed increase of PRL. This increase in proteinuria was also less dramatic than the increase observed in metoclopramide-treated mice (Table 1). Serum concentrations of anti-dsDNA IgG antibody in hyperprolactinemic MRL mice increased 4-fold compared to PBS treated mice (22.96 ± 5.11 and 5.94 ± 1.98 *μ*g/mL, resp.). Similarly, MRL/lpr mice showed increased concentrations in hyperprolactinemic and PBS-treated mice (22.50 ± 5.10 and 11.60 ± 1.20 *μ*g/mL). C57BL/6 mice not presented with proteinuria nor anti-dsDNA antibodies in any condition tested (Table 1). Taken together, these data show that increased PRL concentrations in serum correlates with the early onset of lupus symptoms in lupus-prone mouse strains.

### 3.4. Expression of the Prolactin Receptor in Mice with Hyperprolactinemia

**Figure 3:**
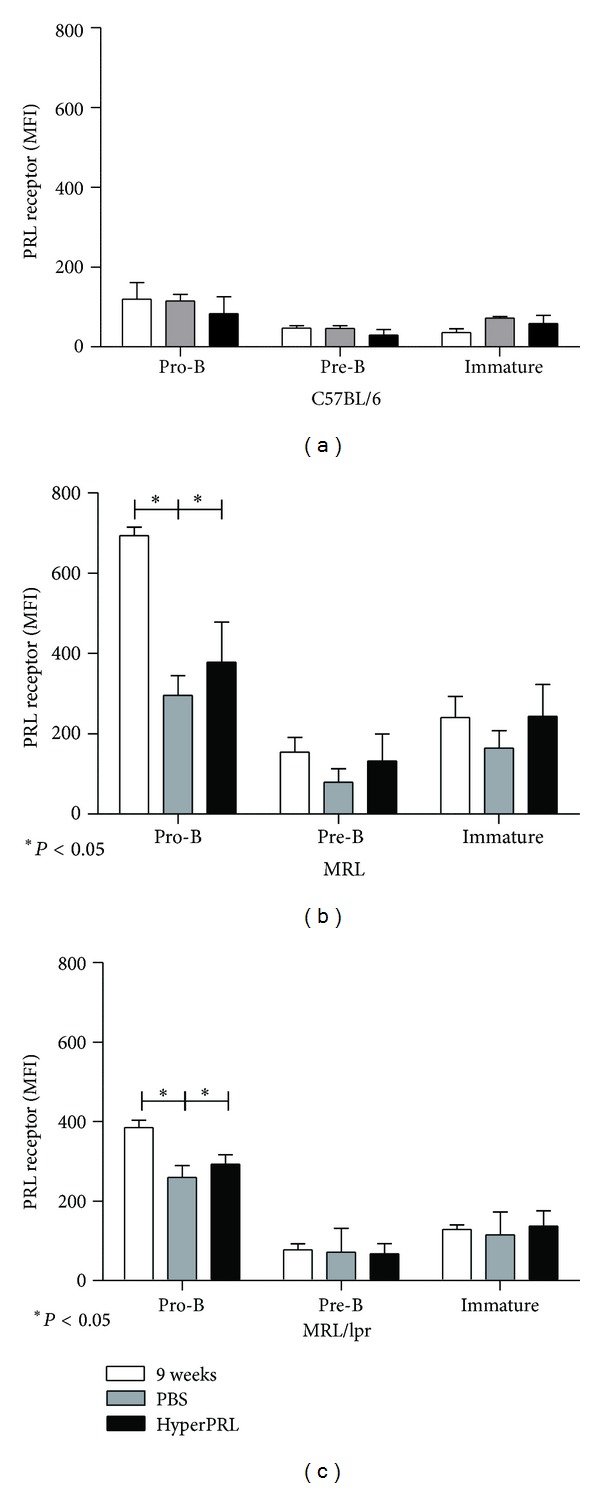
Prolactin receptor expression after the induction of hyperprolactinemia. The levels of PRL receptor protein (MFI) in the B cells from BM (pro-B, preB, and immature) were measured using flow cytometry. At the end of the treatment, the BM cells were labelled with anti-B220, anti-CD43, anti-CD23, anti-IgM, and goat anti-PRL receptor antibodies. (a) C57BL/6 mice; (b) MRL mice; (c) MRL/lpr mice. The asterisks denote statistical significance between populations with the *P* value shown. The MFI values expressed in the graphs correspond to the MFI values minus the isotype control.

We have previously reported that augmented PRL levels in serum result in higher levels of its receptor in B cell splenocytes [20]. When pro-B, pre-B, and immature cells were analysed, we found that hyperprolactinemia did not change PRL receptor expression in the wild-type control strain (Figure 3(a)). Similarly, there were no significant changes between the PBS and metoclopramide treated groups in lupus-prone mice (MRL pro-B cells: 296.00 ± 49.46 and 378.66 ± 79.70 MFI, resp.; pre-B cells: 79.50 ± 33.04 and 132.50 ± 66.96 MFI, resp.; immature B cells: 164.60 ± 43.71 and 221.8 ± 84.03 MFI, resp.; MRL/lpr pro-B cells: 259.80 ± 29.78 and 292.83 ± 59.50 MFI, resp.; pre-B cells: 71.60 ± 27.00 and 67.00 ± 25.98 MFI, resp.; and immature B cells: 115.25 ± 28.63 and 136.60 ± 38.42 MFI, resp.) Figures 3(b) and 3(c). In contrast, an age-related significant decrease (*P* < 0.05) was observed in PBS-treated pro-B cells (MRL = 296.00 ± 49.46, MRL/lpr = 259.80 ± 29.78 MFI) compared with levels before treatment (MRL = 693.60 ± 46.56, MRL/lpr = 385.33 ± 43.78 MFI). This change was larger for MRL than for MRL/lpr mice. Thus, contrary to B cell splenocytes, there is no increase in PRL receptor levels in response to PRL in early B cell populations; instead, there is a decrease in this receptor with age. However, the levels of receptor expression of lupus-prone mice are still significantly higher than those of wild type control mice, especially for pro-B and immature B cells.

### 3.5. Estimation of Population Absolute Numbers

**Figure 4:**
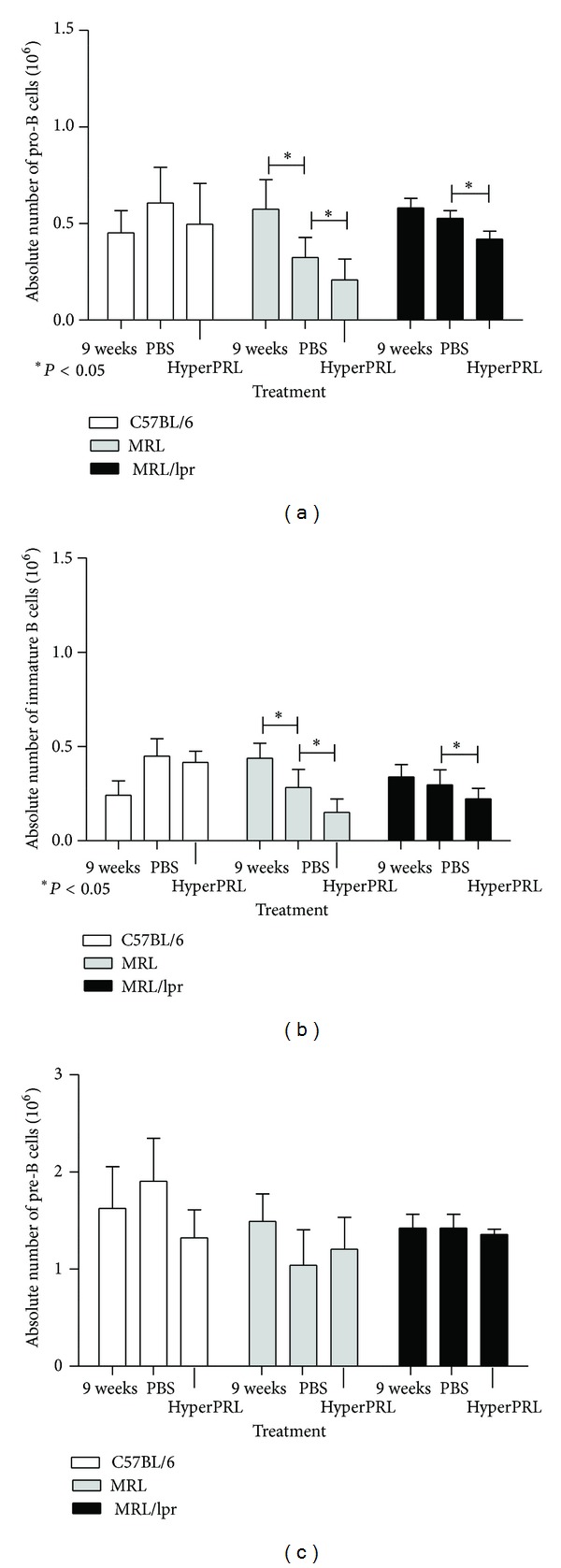
Absolute number of B cells from bone marrow after the induction of hyperprolactinaemia. Nine-week-old mice were treated with metoclopramide (200 *μ*g/100 *μ*L) to induce hyperprolactinaemia (HyperPRL) or PBS (100 *μ*L) for 6 weeks, with eight mice per condition. At the end of the treatment, bone marrow cells were labelled with antibodies against B220, CD43, CD23, and IgM. (a) Graph of the absolute numbers of pro-B cells. (b) Graph of the absolute numbers of immature B cells. (c) Graph of the absolute numbers of pre-B cells. The asterisks denote statistical significance between populations with the *P* value shown.

The absolute cell numbers of bone marrow B cell subsets were analysed as an indicator of possible effects of PRL in B cell development. In C57BL/6 control mice, we did not observe changes in any populations when treated with metoclopramide or PBS. Conversely, mice that developed lupus symptoms had a significant decrease in the absolute number of pro-B cells during hyperprolactinemia (MRL = 0.23 ± 0.11 × 10^6^ cells, MRL/lpr = 0.40 ± 0.05 × 10^6^) compared with PBS-treated mice (MRL = 0.33 ± 0.12 × 10^6^ cells, MRL/lpr = 0.53 ± 0.04 × 10^6^; Figure 4(a)). A decrease was also observed in immature B cells between metoclopramide-treated and PBS-treated mice (MRL = 0.15 ± 0.07 × 10^6^ and 0.30 ± 0.08 × 10^6^ cells, resp.; MRL/lpr = 0.21 ± 0.06 × 10^6^ and 0.31 ± 0.08 × 10^6^ cells, resp.; Figure 4(b)). In contrast, the absolute number of pre-B cells was not affected by the hyperprolactinemic state, as shown in Figure 4(c). Therefore, a decrease in the absolute numbers of pro-B and immature B cells seems to correlate with these cells' basal PRL receptor expression (Figure 2). Although we did not observe further changes in receptor expression during hyperprolactinemia, the data in Figure 4 also support a PRL-mediated effect.

### 3.6. Increase in BIRC5 Expression in Immature Cells

**Figure 5:**
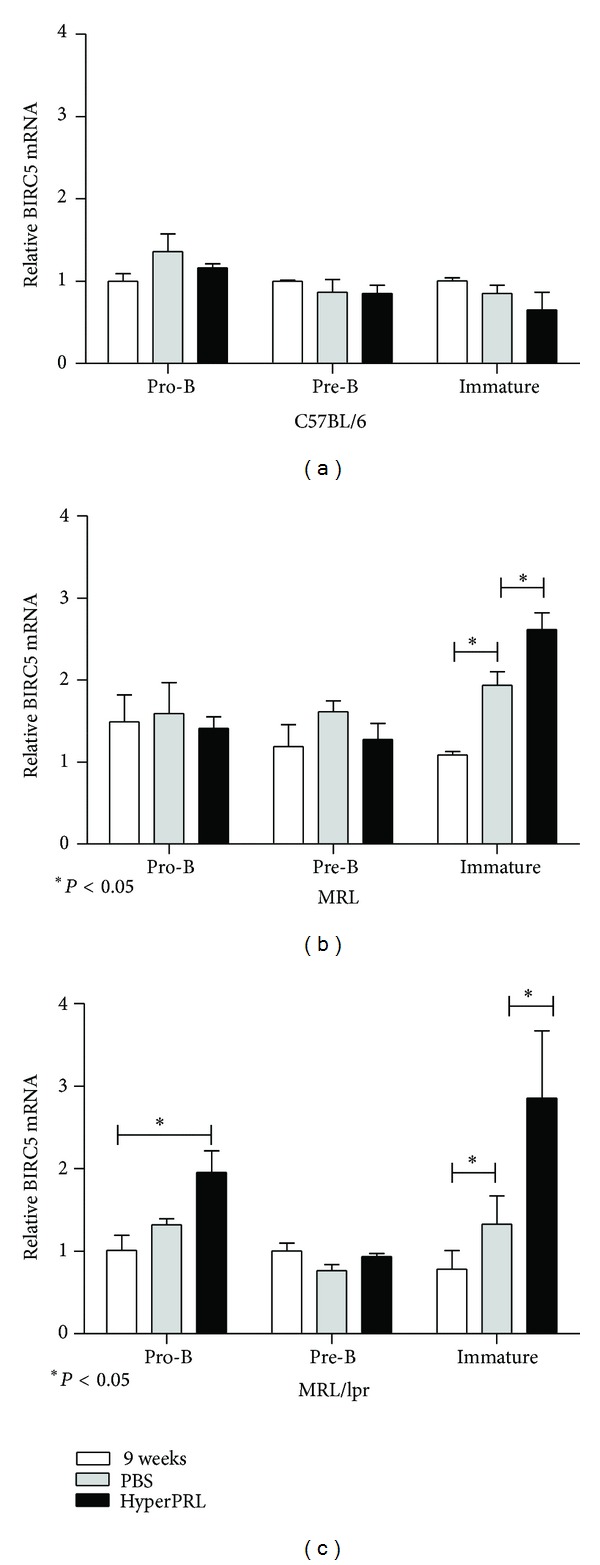
Increase in BIRC5 expression in immature cells. Nine-week-old mice were treated with metoclopramide (200 *μ*g/100 *μ*L) to induce hyperprolactinaemia (HyperPRL) or PBS (100 *μ*L) for 6 weeks. At the end of the treatment, pro-B, pre-B and immature B cells were purified using flow cytometry in three independent experiments using three mice per experiment. Using RT-PCR, the BIRC5 mRNA expression was determined. (a) C57BL/6 mice; (b) MRL mice; (c) MRL/lpr mice. The asterisks denote statistical significance between populations with the *P* value shown.

Contrary to our previous observations in the spleen, numbers of BM B cells with higher levels of PRL receptor were reduced in response to PRL. Because self-reacting immature B cells are selected against, it is possible that PRL mediates the accelerated development of immature B cells. Using a gene expression microarray, we previously observed the upregulation of the BIRC5 gene, a survival factor, in total bone marrow cells from 9-week-old MRL/lpr mice cultured in the presence of PRL (manuscript in preparation). Therefore, we assessed whether the expression of this survival factor is altered during hyperprolactinemia. Bone marrow B cell populations were purified and assayed for BIRC5 expression by real time-RT-PCR.  Figure 5 shows that BIRC5 expression does not vary in the control mice (pretreatment = 1.00 ± 0.03; PBS-treated = 0.85 ± 0.10; and metoclopramide-treated = 0.65 ± 0.21; Figure 5(a)), while expression increased in both lupus-prone strains in response to PRL and aging primarily for immature cells (MRL: pretreatment = 1.08 ± 0.04; PBS-treated = 1.93 ± 0.16; metoclopramide-treated = 2.62 ± 0.20; MRL/lpr: pretreatment = 0.78 ± 0.22; PBS-treated = 1.32 ± 0.24; and metoclopramide-treated = 2.85 ± 0.81; Figures 5(b) and 5(c)) but also for pro-B cells in the MRL/lpr mice (pretreatment = 1.01 ± 0.18; PBS-treated = 1.32 ± 0.07; and metoclopramide-treated = 1.95 ± 0.26). Therefore, a correlation also exists between the expression of the survival factor BIRC5 and basal levels of the PRL receptor. Upregulation of antiapoptotic genes in immature B cells in response to PRL levels may be an important mechanism of escaping tolerance mechanisms and may explain the increase in autoantibodies in PRL-triggered SLE.

## 4. Discussion

Several studies have demonstrated the importance of PRL and B lymphocytes in the development of SLE [12–16]. We previously reported that all subsets of splenic B cells (T-1, T-2, T-3, follicular and marginal zone cells) express the PRL receptor, with the highest expression in the most immature subset (T-1s) in the lupus-prone mouse strains [20]. Because T-1s are directly produced from B cells developing in the bone marrow, we evaluated the expression of the PRL receptor in different bone marrow developmental stages (pro-B, pre-B and immature) as well as the response of these populations to the pharmacological induction of a hyperprolactinemic state and correlated our observations with the course of SLE in MRL/lpr, MRL, and wild-type mice (C57BL/6). We found that bone marrow B cells also express the PRL receptor in all of the mouse strains analysed. However, in wild type mice, the expression of the receptor decreases as the B cell matures, while in strains that develop SLE, immature B cells together with pro-B cells have significantly higher levels of the PRL receptor compared to pre-B cells and their wild-type counterparts. The fact that the PRL receptor is expressed throughout all stages of the B cell developmental pathway supports the important role of PRL in B cell maturation and therefore in the function of B cell effectors.

It is known that increased PRL levels favour the appearance of SLE manifestations in NZB × NZW [28], *Sle3/5* R4A-*γ*2b C57BL/6 [29] and MRL/lpr mice [20]. In MRL and MRL/lpr strains, hyperprolactinemia correlates with premature SLE manifestations as well as increased receptor expression and aberrant B cell development in the spleen [20]. Although we did not observe differences in PRL receptor expression in response to PRL, we observed a reduced number of pro-B, and immature B cells with a high basal PRL receptor expression, indicating a PRL-mediated effect on B cell development. These data support an association between BM B cell maturation and disease progression. This observation is also supported by reports in which the SLE-prone strains MRL/lpr [30], BXSB [31], and NZB [32, 33] presented age-related anomalies in B cell development that were correlated with disease manifestations [34]. 

The main goal of B cell early development is to generate a functional BCR that is not self-reactive and B cell maturation is strongly dependent on either constitutively generated (tonic) signalling or ligand-induced BCR signalling [35]. Increased levels of IL-7 or the ectopic expression of antiapoptotic genes have resulted in the increased proliferation and survival of developing cells, but progression is arrested in the absence of these signals [36]. To our knowledge, there have been no previous *in vivo* studies of the effect of PRL on early B cell development. The reduced numbers of pro-B and immature B cells observed in response to PRL could be due to either accelerated developmental progression or increased negative selection. 

Sexual hormones, such as oestrogens, regulate lymphopoiesis; pro-B lymphocytes are especially sensitive to high oestrogen concentrations, resulting in decreased numbers of these cells, where oestrogen can arrest lymphoid lineage differentiation [37, 38]. A similar PRL effect may explain the reduced numbers of pro-B cells observed during hyperprolactinemia. We also observed decreased numbers of immature B cells, which is an interesting observation because this is one of the main populations subjected to regulation against self-recognition. In transgenic mice in which BCR survival/tonic signals are favoured, for example, the SHIP knockout mice, accelerated development resulting in decreased immature/transitional populations has been observed [39]. PRL-triggered accelerated development throughout the immature stage would also explain our previous observation of the accumulation of T1 B cells in the spleen [20]. PRL may counteract mechanisms that prevent the self-reactivity of immature B cells, facilitating their rapid exit from bone marrow and the feeding of the splenic T-1 pool. Therefore, the results by Morales et al. [40] regarding PRL-induced B cell development argue for B cell maturation coordinated by the BCR together with environmental signals. These latter signals, such as PRL-induced signals, are also critical and may shape the B cell repertoire in response to different physiological stages.

Ligand-induced BCR signals are often associated with triggering the elimination of autoreactive clones at immature and transitional stages. PRL receptor signalling is known to increase the expression of antiapoptotic genes, such as Bcl-2 [41, 42], and T-1 B cells from hyperprolactinemic BALB/c mice are more resistant to apoptosis [43]. In line with these observations, we found that PRL increases the expression of the BIRC5 (survivin) gene (see Supplementary Figure 1 in supplementary material available online at http://dx.doi.org/10.1155/2013/287469), which belongs to a family of apoptosis inhibitors (IAP) [44, 45]. Survivin plays an important role in cell cycle entry/progression, maturation, and the inhibition of apoptosis as well as increasing the survival of hematopoietic stem/progenitor cells [46–50]. An increased expression of BIRC5 in immature B cells was found only in the SLE mice in response to hyperprolactinemia. Furthermore, BM B cells incubated with an anti-IgM antibody have increased survival rates in hyperprolactinemic conditions (Supplementary Figure 2). Taken together, these data indicate an important effect of PRL on B cell development, both favouring positive selection and counteracting mechanisms against self-specificity. In this scenario, increased PRL levels would result in the maturation of B cell clones with self-reactivity and an increased risk for developing autoimmune diseases. It will be interesting to determine the molecular mechanisms by which PRL and PRL receptors interfere with B cell maturation and tolerance, which will aid in the rational design of targeted therapy with potential applications for both autoimmunity and immunodeficiencies.

## 5. Conclusions

The PRL receptor is expressed by pro-, pre-, and immature B cells in the bone marrow suggesting an important role for PRL in early B cell development. In agreement, both populations with increased receptor expression, pro-Bs and immatures, upregulate the expression of survival factor BIRC5 in response to PRL. This might be an important mechanism for breakdown of tolerance, since PRL-enhanced BIRC5 expression correlated with an early onset of lupus symptoms.

## Supplementary Material


**Supplementary Figure 1. Increase in BIRC5 expression.** B cells were purified from the BM of 9-week-old mice and incubated with medium and PRL (50 ng/ml) at different times. Using real time RT-PCR, BIRC5 mRNA expression was determined. (a) C57BL/6 mice; (b) MRL mice; (c) MRL/lpr mice. The asterisks denote statistical significance between populations with the *P* value shown.
**Supplementary Figure 2. Prolactin increased the survival in B cells.** B cells were purified from the BM of 9-week-old mice and incubated with: (i) medium, (ii) antibody anti-IgM (10 *μ*g/ml) and (iii) antibody anti-IgM plus PRL (50 ng/ml) for 24 h. At the end of the incubations, the cells were labelled with DAPI to count the live cells (DAPI−). (a) C57BL/6 mice; (b) MRL mice; (c) MRL/lpr mice. The asterisks denote statistical significance between populations with the *P* value shown.

## Figures and Tables

**Table 1:** SLE manifestations in mice with hyperprolactinemia.

Strain	C57BL/6			MRL			MRL/lpr		
Treatment	9 weeks	PBS	HyperPRL	9 weeks	PBS	HyperPRL	9 weeks	PBS	HyperPRL
PRL (ng/mL)	4.2 ± 1.3	3.8 ± 1.1	10.7 ± 1.2*	12.5 ± 1.9	11.2 ± 1.8	26.27 ± 2.9*	12.7 ± 2.2	20.0 ± 2.7*	34.5 ± 4.3*
Proteinuria (mg/mL)	0	0	0	10.1 ± 7.2	12.8 ± 5.6	121.6 ± 37.4*	13.5 ± 8.5	48.0 ± 18.6*	166.6 ± 23.5*
Ab anti-dsDNA (*μ*g/mL)	0	0	0	0	5.9 ± 1.9*	22.9 ± 5.1*	2.5 ± 0.05	11.6 ± 1.2*	22.5 ± 5.1*

HyperPRL: hyperprolactinemia.

*ANOVA, *P* < 0.05.
