# Supplementary figures and images for: Prolactin Levels Correlate with Abnormal B Cell Maturation in MRL and MRL/lpr Mouse Models of Systemic Lupus Erythematosus-Like Disease
Source: Clin Dev Immunol. 2013 Dec 10;2013:287469. doi: 10.1155/2013/287469 (PMC3878598; doi:10.1155/2013/287469)

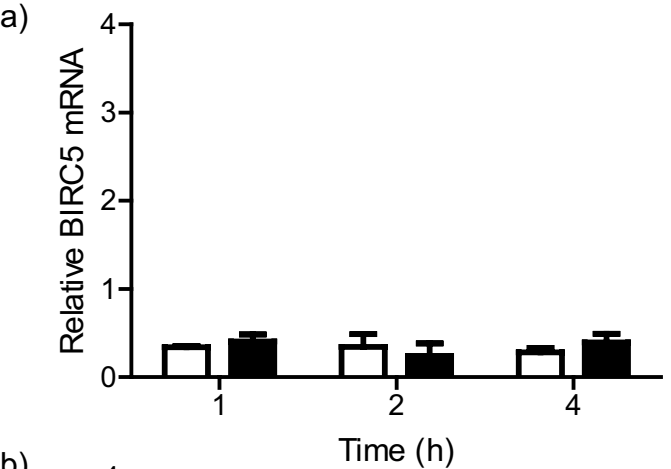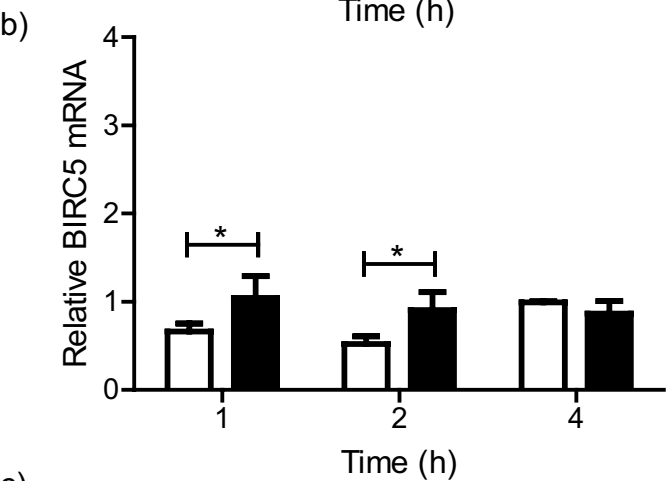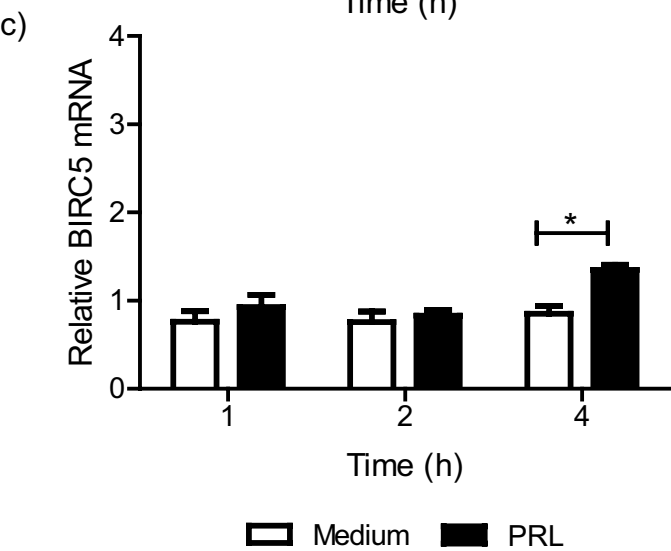

Supplement: Supplementary file 1 — Supplementary Figure 1. Increase in BIRC5 expression. B cells were purified from the BM of 9-week-old mice and incubated with medium and PRL (50 ng/ml) at different times. Using real time RT-PCR, BIRC5 mRNA expression was determined. (a) C57BL/6 mice; (b) MRL mice; (c) MRL/lpr mice. The asterisks denote statistical significance between populations with the P value shown. Supplementary Figure 2. Prolactin increased the survival in B cells. B cells were purified from the BM of 9-week-old mice and incubated with: (i) medium, (ii) antibody anti-IgM (10 μg/ml) and (iii) antibody anti-IgM plus PRL (50 ng/ml) for 24 h. At the end of the incubations, the cells were labelled with DAPI to count the live cells (DAPI−). (a) C57BL/6 mice; (b) MRL mice; (c) MRL/lpr mice. The asterisks denote statistical significance between populations with the P value shown. [file 287469.f1.pdf]

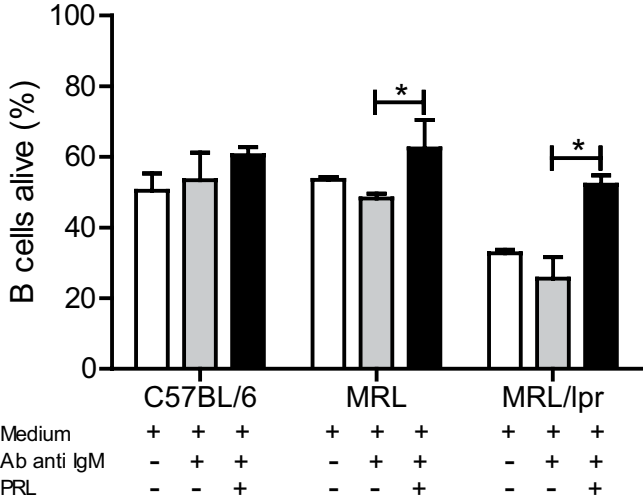

Supplement: Supplementary file 2 [file 287469.f2.pdf]
